# Phase Diagram of Dairy Protein Mixes Obtained by Single Droplet Drying Experiments

**DOI:** 10.3390/foods11040562

**Published:** 2022-02-16

**Authors:** Ming Yu, Cécile Le Floch-Fouéré, Jeehyun Lee, Françoise Boissel, Romain Jeantet, Luca Lanotte

**Affiliations:** STLO, INRAE, Institut Agro, 35042 Rennes, France; ming.yu@inrae.fr (M.Y.); cecile.lefloch-fouere@agrocampus-ouest.fr (C.L.F.-F.); jeehyun.lee@agrocampus-ouest.fr (J.L.); francoise.boissel@agrocampus-ouest.fr (F.B.); romain.jeantet@agrocampus-ouest.fr (R.J.)

**Keywords:** drying, dairy proteins, phase diagram

## Abstract

Dairy powders are mainly produced by droplet spray drying, an articulated process that enables the manufacture of high added-value goods with a long shelf life and well-preserved functional properties. Despite the recent advances, a full understanding of the mechanisms occurring at the droplet scale in drying towers and, consequently, of the impact of process parameters and processed fluid characteristics on the powder properties is far from being achieved. In the wake of previous studies based on a laboratory scale approach, in this work, we provided a global picture of the drying in droplets of dairy protein mixes, i.e., whey proteins and casein micelles, which represent crucial dairy powder ingredients. Using profile visualization and optical microscopy, we explored the shape evolution in droplets with a range of protein contents and compositions typical of commercial powder production. The observation favored the evaluation of the specific role of each protein on the evaporation dynamics, and led to the construction of a phase diagram predictive of the dry droplet shape starting from the characteristics of the initial protein dispersions. Our outcomes represent a further step shedding light on the paradigm linking the physics of drying at the microscale and the nutritional properties of complex dairy powders.

## 1. Introduction

In the last few decades, the dairy industry has had to adapt to the rapid changes of market trends, resulting in a continuous evolution in terms of production efficiency and design of innovative processing techniques and products. Indeed, the rapid economic improvement of developing countries (e.g., China, India, and Brazil) and the constant world population growth has led to so-called globalization and, consequently, to the necessity of producing goods for end users all over the world, i.e., with a relatively long shelf life, being easy to transport and handle, and at the same time preserving the controlled functional properties. Dairy powders are a glaring example of this category of products and are nowadays used not only as a substitute for milk, but also to formulate specialized nutrition for athletes, the elderly, and most of all, for infants (infant milk formulas (IMFs)) [1]. IMFs have the main goal of feeding infants up to 12 months old and, hence, have to mimic the evolving nutritional properties of human breast milk. This objective is quite challenging due to the high complexity of the human milk system from the physico-chemical point of view, and as the main raw material employed for IMF production, i.e., cow milk, has a significantly different composition than human milk.

IMFs are obtained after a processing sequence of industrial unit operations culminating in spray drying towers, where microscopic droplets of dairy fluids (≈100–400 µm) transform into solid particles as a result of fast mass and energy transfer phenomena (time scale ≈ 4–5 s) [2]. Over the years, both theoretical and experimental works have shown that environmental conditions and droplet characteristics (i.e., solute concentration and composition) strongly affect the droplet-to-particle transition and, furthermore, powder functional properties [3,4] (e.g., rehydration [5] and sticking behavior [6]). Thus, tight control of the drying process and its key parameters would allow for tuning the characteristics of the final product while reaching a specific nutritional target. However, despite recent technological advances, typical spray drying chambers remain “black boxes” from a physical point of view, and a full understanding of the mechanisms occurring during the fast phase transition at the droplet scale is far from being achieved. For this reason, numerous research groups have adopted alternative methods in the last decade, especially at the laboratory scale [7,8,9], to investigate the drying dynamics in droplets of different dairy liquids (e.g., whole milk, skim milk [10], and dairy protein mixes [11,12,13]). Considering the net of the differences in terms of the space and time scale with respect to the industrial dryers, the observation of single-droplet behavior favored the detection of the main stages of the evaporation process and the preliminary evaluation of the impact of different milk components on their occurrence and development [9,14,15]. Therefore, these works represent a pioneering link between the empirical experience of industrial technology and the study of the physics of biological fluids.

In the case of mixes of dairy proteins (i.e., the whey proteins and the casein micelles), which are one of the main components of milk and are crucial IMF ingredients, the study of droplet drying at the microscale fits into the wider and more complex context of the evaporation in polydisperse colloidal systems [16,17,18]. Whey proteins (average diameter of δ = 10–30 nm) are characterized by a rigid globular shape [19], whereas casein micelles (δ = 100–300 nm) exhibit a typical deformable sponge-like structure [20]. Therefore, due to their different physico-chemical properties, previous works have focused on the investigation of the possible separate role of each of these biocolloids in the development of the drying stages, i.e., (i) droplet homogeneous shrinkage, (ii) interfacial sol−gel transition and surface solidification, and (iii) evaporation of the remaining solvent without morphological changes. In particular, the sol−gel transition at the air−liquid interface, leading to the formation of the so-called skin or crust, has been systematically explored in droplets of dairy protein mixes [9,12,13] looking for the eventual “signature” of the molecular scale on the final particle shape [11,21]. Most of these observations were performed on pendant or sessile droplets of whey proteins (in the form of whey protein isolates (WPI)) and casein micelles (in the form of native phosphocaseinates (NPC)), visualizing their profile evolution throughout the evaporation or by optical microscopy. At an early stage, the seminal works of Sadek et al. on single protein drying droplets showed that the main colloidal properties of WPI and NPC can be recognized in the morphology of the dry particles: rigid, smooth, and hollow in the case of WPI suspensions [9], and wrinkled and invaginated for NPC samples [15]. In the wake of these promising outcomes, the experimental activity was also directed to WPI/NPC mixtures. For example, Lanotte et al. illustrated that, more than a hybrid shape reflecting the simultaneous influence of both proteins, dry WPI/NPC particles exhibited the dominant morphological characteristics of one biocolloid depending on the protein ratio in the initial dispersion [12]. This suggested a possible protein preferential segregation at the droplet surface induced by the evaporation, which would influence the rheological and mechanical properties of the skin. This hypothesis was corroborated in the same work performing the elemental analysis of dry WPI/NPC shells, and was then further confirmed by Yu et al. with the observation by the scanning electron microscopy (SEM) of the dry section of WPI/NPC droplets [13]. These studies highlighted the accumulation of the whey proteins on the skin external layer (i.e., small-on-top) [22,23], and the presence of casein micelles was detected predominantly in the core of the particles.

All in all, these studies strengthened the assumption that it would be possible to predict the dry particle shape and thus functional properties in dairy protein mixes knowing the overall protein concentration (c_p_) and WPI/NPC ratio in the initial dispersions. In this work, we propose the first phase diagram predictive of WPI/NPC dry particle morphology as a function of these two key parameters, ranging in the values typical of industrial plants for commercial powder production. We performed further tests of profile visualization and optical microscopy in top view on drying WPI/NPC droplets to complete the global picture provided by the previous outcomes present in the literature. We also interpreted the impact of c_p_ and sample composition on the droplet shape evolution in the light of the possible physical events occurring at the droplet air−liquid interface throughout the evaporation process.

## 2. Materials and Methods

### 2.1. Sample Preparation

Dispersions of single WPI and NPC powders with an overall protein concentration (c_p_) ranging from 6 wt% to 14 wt% were prepared by dissolving WPI and NPC commercial powders (protein content equal to 86 wt% and 82 wt%, respectively) in deionized water containing 0.02 wt% of sodium azide (NaN_3_) as a bacteriostatic agent. The suspensions were stirred for 48 h at room temperature (T = 20 °C) to ensure full dissolution. Afterward, WPI/NPC mixtures with different composition were obtained, for each fixed c_p_, by mixing the single protein dispersions and regulating the WPI relative percentage (WPI%_R_), defined as follows (Equation (1)):

(1) $${\mathrm{WPI}\%}_{R}=\frac{m_{\mathrm{WPI}}}{m_{\mathrm{solute}}}$$

where m_WPI_ is the mass of WPI and m_solute_ refers to the sum of WPI and NPC powders in the suspensions. In this work, WPI%_R_ was adjusted to 0, 20, 50, 80, or 100%. Due to the different voluminosity of WPI and NPC macromolecules, which was equal to 0.74 mL·mg^−1^ [24] and 4.4 mL·mg^−1^, respectively [25], the final WPI/NPC mixes exhibited significant differences in terms of volume fraction (φ_p_), as summarized in Table 1, as a function of c_p_ and WPI%_R_.

### 2.2. Single Droplet Experiments

The droplets of WPI/NPC mixtures (V_0_ = 0.5 µL) were deposited on a poly dimethyl siloxane (PDMS) surface characterized by micrometric pillars, as illustrated in detail in previous works [9]. Such a patterned hydrophobic support reduced the contact surface and conferred to the droplets an almost hemispherical shape (initial contact angle ≈ 105°) at the early stage of the evaporation, regardless of c_p_ and WPI%_R_. Then, the setup was rapidly transferred into a sealed glass box equipped with zeolites (HG2-DES-3, Rot) to minimize the relative humidity (RH ≈ 2%). The environmental temperature was kept constant at 20 ± 1 °C.

The observation of the droplet shape evolution with time was realized by profile visualization and in top view. The profile history was recorded (fps = 0.2) with a high-speed camera (Fastcam MC2 10,000 NB, Photron). A light source (Phlox 100/100 LLub) was placed in the opposite direction of the camera to get a uniform background and to enhance the quality of the image contrast. Regarding the top view observation, the morphology was monitored with an optical microscope (Olympus BX51TF) equipped with a QIClick^TM^ Digital CCD Camera (fps = 1). The experiments were repeated ten times for each sample.

### 2.3. Measurement of the Main Shape Parameters

**Figure 1:**
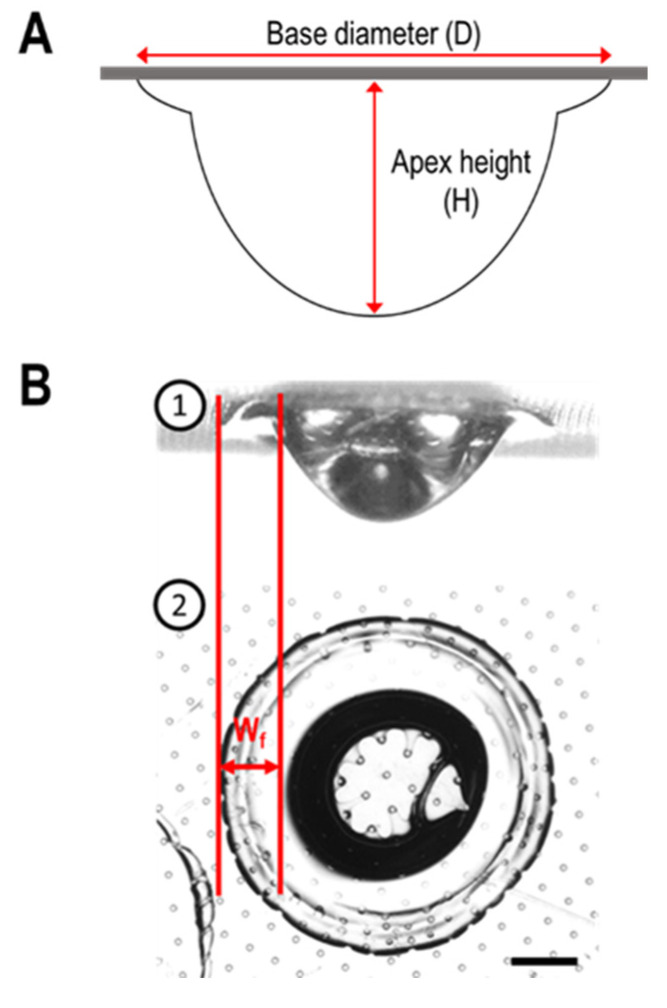
(**A**) Definition of the base diameter (D) and apex height (H) in drying WPI/NPC droplets. (**B**) Foot width (w_f_) observed by profile visualization ➀ and top view ➁ at the end of the evaporation in a WPI droplet. Scale bar equal to 200 µm.

The image sequences acquired throughout the evaporation were analyzed offline by a custom image analysis software (ImageJ) to measure the variation with time of the droplet shape key parameters. The profile view approach allowed for characterizing the variation with the time of the base diameter (D), i.e., the average diameter of the contact surface between the droplet and substrate during the drying process, and the apex height (H), i.e., the maximum distance between the droplet apex and substrate, for all WPI/NPC droplets (Figure 1A), in agreement with previous works on the drying of dairy protein dispersions [9,12,13,15]. On the other hand, the top view observation favored the estimation of the foot width (w_f_) as a function of c_p_ and WPI%_R_ at the end of the evaporation process (Figure 1B).

The diameter reduction induced by the drying process was defined as (Equation (2)):

(2) $$\Delta D\%=\frac{\left(D_{\max}-D_{f}\right)}{D_{\max}}$$

where D_max_ and D_f_ refer to the maximum and final base diameter, respectively (N.B., D_max_ ≠ D_0_, i.e., the initial value, as droplets often slightly expand during the early drying stage).

## 3. Results and Discussion

### 3.1. Quantification of Base Diameter and Apex Height in WPI Droplets

**Figure 2:**
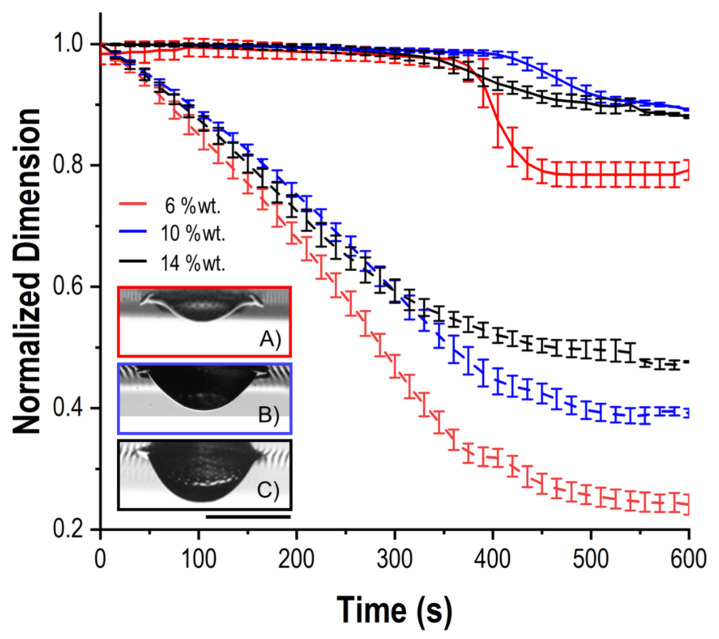
Evolution of the normalized droplet base diameter (D/D_max_, solid line) and apex height (H/H_max_, dashed line) with time for WPI samples with different protein concentrations (c_p_). In the inset, pictures of dry WPI particles with c_p_ = 6 wt% (**A**), 10 wt% (**B**) and 14 wt% (**C**) are illustrated. Scale bar equal to 500 µm.

The systematic analysis of D and H by profile observation provided indirect evidence of the impact of the sample characteristics (c_p_, WPI%_R_) on the droplet morphology changes induced by the evaporation, here quantified by the diameter reduction and the final apex height. In Figure 2, the evolution of D (solid line) and H (dashed line) normalized by their maximum values (D_max_, H_max_) is displayed, as a reference, for WPI suspensions (WPI%_R_ = 100%) with different c_p_, as in these samples, the shape modifications are more evident than in other mixes. For the sake of clarity, only the data related to c_p_ = 6 wt%, 10 wt% and 14 wt% are shown. As it concerns the D/D_max_ curves, a transition from border detachment (Figure 2A) to slight shrinkage (Figure 2B), similar for all WPI droplets when c_p_ ≥ 10 wt%, was observed with increasing the initial protein content in the suspensions. The attenuation of the delamination at a higher c_p_ could be explained by the enhanced accumulation of solutes driven by drying-induced outward capillary flows [26,27]. This would lead to the formation of a larger external “foot” able to withstand the surface inward stresses due to its structural robustness and possibly even its improved chemical affinity with the substrate. Indeed, in the Materials and Methods section, the respective protein content has been reported for the WPI and NPC powders used for the sample preparation. This means that these commercial powders contained a minor amount (5–6 wt%) of the other protein, as well as of lactose and minerals. In previous studies, NPC colloids have been proven to favor the triple line adhesion in drying droplets, resulting in negligible border detachment [15]. Therefore, it is likely that, for c_p_ ≥ 10 wt%, WPI dispersions include enough casein micelles contributing to the foot anchoring to the PDMS surface. On the other hand, increasing c_p_ also corresponded to a gradually higher final apex height. Such a behavior could be interpreted in the light of the probable earlier achievement of the surface sol−gel transition conditions, i.e., for a WPI critical concentration c_gel_ = 41 wt%, resulting in the development of a thicker skin layer [9].

### 3.2. Diameter Reduction

If the outcomes obtained for pure WPI droplets led to a linear interpretation, the investigation appeared more complex in the case of WPI/NPC mixes, where the coexistence of colloids with different sizes, structures, and mechanical properties resulted in possible competitive dynamics. To avoid the redundancy of presenting the evolution of D/D_max_ and H/H_max_ for all WPI/NPC dispersions, we focused here on the diameter reduction (ΔD%) and the final apex height (H_f_) as a function of WPI%_R_ in droplets with different c_p_.

**Figure 3:**
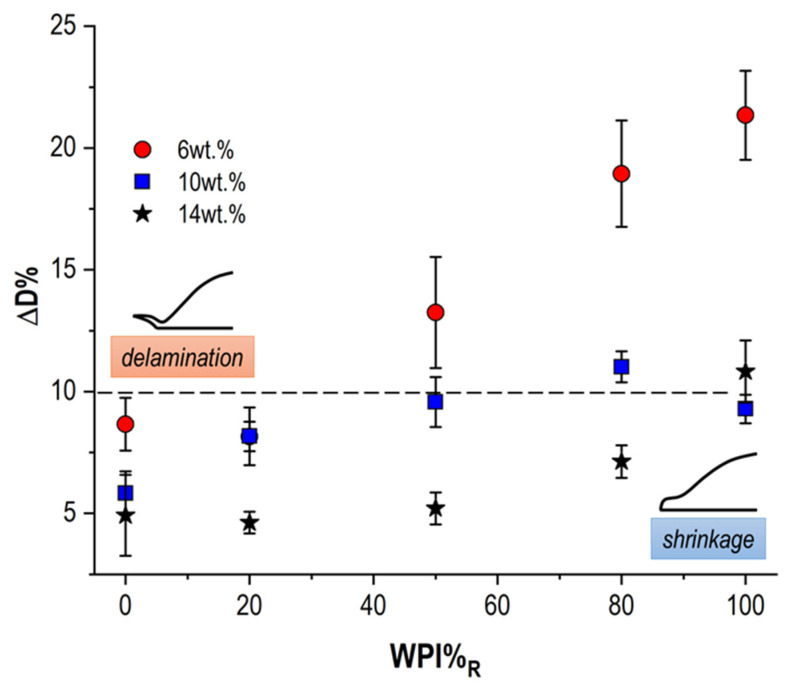
Estimation of the diameter reduction (ΔD%) as a function of WPI%_R_ at the end of the drying process in WPI/NPC droplets with c_p_ = 6 wt%, 10 wt%, and 14 wt%.

For the sake of clarity, the ΔD% values related to all the explored WPI/NPC mixes are reported in Table 2, whereas in Figure 3 only the outcomes for suspensions with c_p_ = 6 wt%, 10 wt%, and 14 wt% are displayed. The outcomes highlighted that an increasing amount of whey proteins in the binary mix corresponded to a more significant diameter reduction, resulting in delamination (ΔD% ≥ 10%) under certain conditions (c_p_ ≤ 8 wt%). This is not surprising, as numerous works in the literature have shown that, contrary to what already mentioned for NPC, the brittle character of WPI-rich dry structures fosters border delamination [9,12]. However, the increase in the overall protein concentration produced strong mitigation of the diameter reduction, until a ΔD% < 10% for c_p_ ≥ 10 wt% regardless of WPI%_R_. This corroborates the hypotheses of a possible thicker foot width and enhanced adhesivity with increasing c_p_.

**Figure 4:**
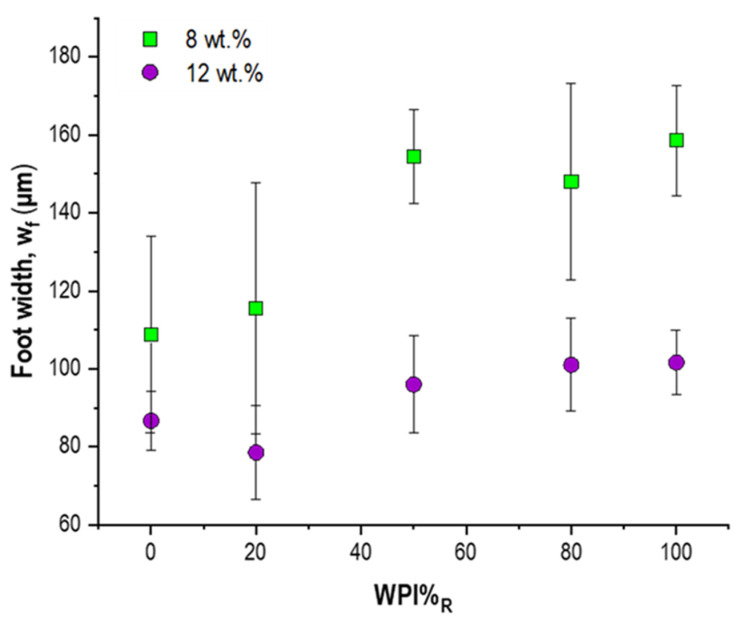
Measure of droplet foot width (w_f_) as a function of WPI%_R_ for c_p_ = 8 wt%, and 12 wt%.

Thus, to investigate the potential dependency of the droplet foot width (w_f_) on c_p_, we performed a quantitative analysis of w_f_, as already described in Figure 1B, as a function of WPI%_R_ for c_p_ = 8 wt% (i.e., droplets exhibiting delamination) and c_p_ = 12 wt% (i.e., samples displaying slight shrinkage). The results illustrated in Figure 4 highlight once more the strong impact of WPI%_R_ on the shape characteristics of drying WPI/NPC droplets. Indeed, we observed an increasing w_f_ with a higher WPI%_R_, especially in the case of c_p_ = 8 wt%. This result was quite surprising, as, with the same mass concentration in the sample, the NPC colloid average size and, consequently, volume fraction in the dispersion were much higher than the WPI ones. As a consequence, a thicker width in NPC-rich droplets should be expected. In addition, and even more surprisingly, droplets with c_p_ = 12 wt% exhibited, on average, a smaller foot width compared to those with a lower overall protein concentration. A possible explanation for these outcomes could be found in the intensity of the internal flows during the evaporation [28]. In fact, casein micelles are characterized by a slower diffusion than whey proteins, due to their size and their different structures [13]. A lower NPC mobility would lead, on the one hand, to early gelation at the surface (evaporation front retraction faster than colloid Brownian motion) [11,15] and, on the other, to the development of weaker capillary flows. In this light, it would be possible to predict the formation of a smaller foot for low WPI%_R_. This effect would even be emphasized by the increase of c_p_, due to the increase of NPC concentration and colloid crowding events, in general, which would further slow down the internal motions in the droplets.

All in all, the outcomes of Figure 4 underline that the attenuation of the delamination phenomena at a higher c_p_ is not linked to the formation of an external foot with a larger size. Therefore, rather than w_f_, NPC adhesivity to the PDMS substrate could be the most plausible reason for the inhibition of border detachment at the end of the drying process. In fact, starting from the overall φ_p_ reported in Table 1 and considering the casein micelle voluminosity, it is possible to estimate that the NPC volume fraction would be higher than the WPI one, even when WPI%_R_ = 80% (e.g., φ_NPC_ = 0.055 for c_p_ = 6 wt%, and φ_NPC_ = 0.12 for c_p_ = 14 wt%). It is worth pointing out that the values of φ_p_ and φ_NPC_ were calculated starting from the ideal assumption of pure initial powders of WPI and NPC, thus without taking into account any minor component. Furthermore, the mechanical response to the inward interfacial stresses occurring during the evaporation, which are the main cause of the diameter reduction, is related to onset of the sol−gel transition at the droplet surface. Therefore, this is also related to the thickness and the structure of the skin.

### 3.3. Droplet Final Apex Height

**Figure 5:**
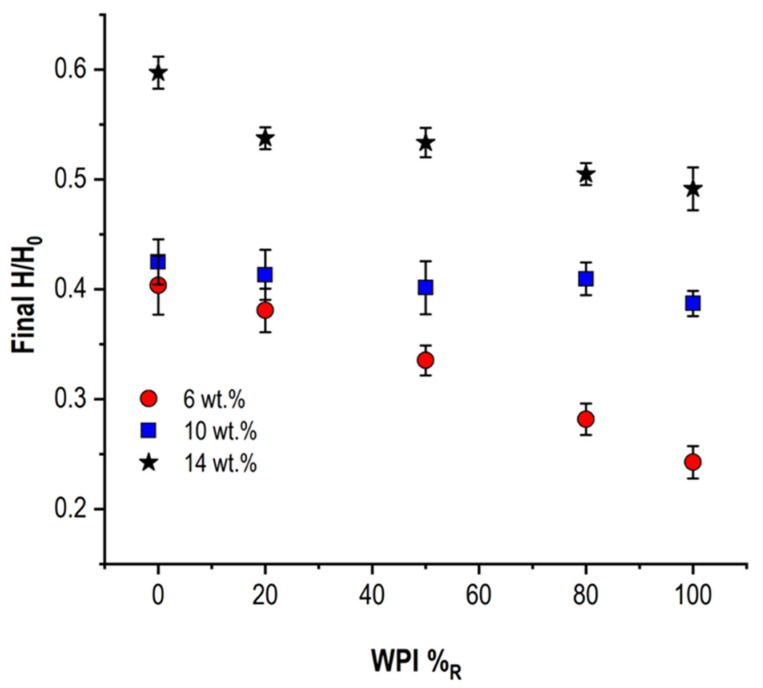
Evaluation of the impact of WPI%_R_ on WPI/NPC droplet final normalized height (H_f_/H_max_) for dispersions with c_p_ = 6 wt%, 10 wt%, and 14 wt%.

In Table 2, the data referring to H_f_ normalized by their maximum values (H_f_/H_max_) are reported for WPI/NPC droplets as a function of c_p_ and WPI%_R_. For the sake of clarity, H_f_/H_max_ values as a function of WPI%_R_ are illustrated in Figure 5 for c_p_ = 6 wt%, 10 wt%, and 14 wt%. This kind of analysis did not provide a characterization of skin morphology at the end of the drying (e.g., buckling instabilities), but rather an evaluation of the impact of sample characteristics (c_p_ and WPI%_R_) on the occurrence of the gelation at the droplet surface, and, therefore, of skin development. For a low c_p_ (≤8 wt%), a significantly lower H_f_/H_max_ was measured with increasing WPI%_R_, with a difference between NPC and WPI samples equal to 0.25. Such an evident tendency was in agreement with the hypothesis of later interfacial sol−gel transition in the presence of larger amounts of whey proteins. A similar, but mitigated, trend was observed in more concentrated samples (c_p_ ≥ 12 wt%), where the difference between H_f_/H_max_ in NPC and WPI droplets was reduced to 0.05–0.07. These results could be interpreted once more in the light of a higher concentration of NPC micelles in the dispersions, thus fostering the occurrence of an earlier sol−gel transition compared to more dilute suspensions. Finally, unexpected behavior was detected for the intermediate c_p_ = 10 wt%, where the measured values of H_f_/H_max_ exhibited a plateau tendency irrespective of WPI%_R_.

A possible interpretation of these outcomes could lie in the evolution of the mechanical properties of the skin and its reaction to the surface instabilities, leading to the buckling in samples rich in casein micelles and to delamination in WPI-rich droplets. However, a systematic study of skin characteristics as a function of solute concentration and composition is currently only at an “embryonic” stage. Indeed, the skin thickness (δ_s_) has already been measured by the authors using SEM in WPI droplets with different c_p_. The subsequent step will be to enrich these first experiments with further tests on WPI-rich samples (i.e., WPI%_R_ = 50%, 80%). This would allow for evaluating the impact of NPC on colloid self-arrangement and final δ_s_, mostly in light of the outcomes recently published on the drying-induced stratification in similar protein mix systems. It would not be likely to perform the same characterization on dispersions with WPI%_R_ ≤ 20%, as in this case the droplets display a very irregular and wrinkled surface, and the cutting would be, consequently, quite complicated in this case.

### 3.4. Phase Diagram of Dried WPI/NPC Droplets

**Figure 6:**
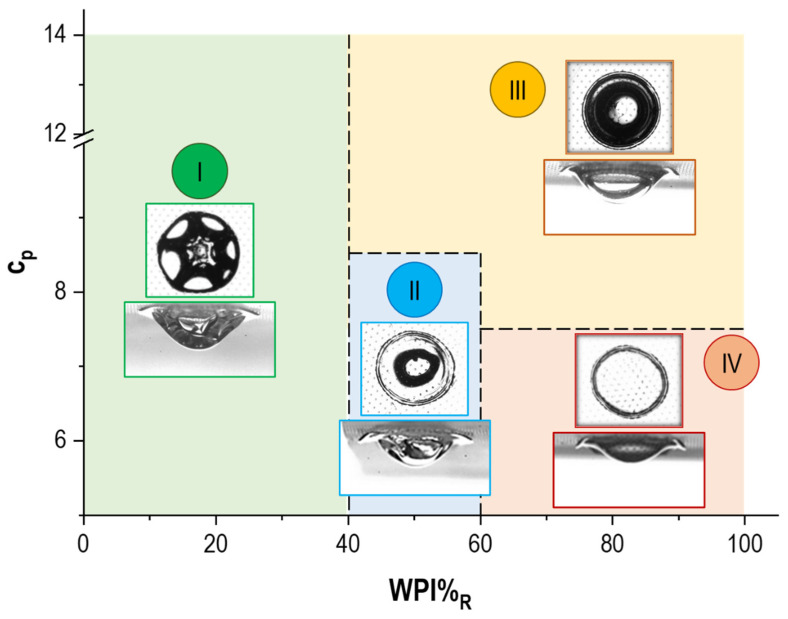
Phase diagram of the main shape characteristics of dried WPI/NPC droplets as a function of the protein overall concentration (c_p_) and sample composition (WPI%_R_). (**I**) Wrinkled surface, no border detachment, and vacuole formation. (**II**) Hybrid shape, significant delamination, vacuole formation. (**III**) Smooth interface, border detachment, and vacuole formation. (**IV**) Smooth surface, significant delamination, and no vacuole formation.

The full characterization of the droplet base diameter (D) and apex height (H), coupled with the observation of the vacuole formation at the late stage of the evaporation process [9,29,30], led to the construction of a preliminary phase diagram for mixes of whey proteins and casein micelles. The goal of the latter is mainly to predict the characteristic shape of dry WPI/NPC particles, knowing the initial protein concentration (c_p_) of the dispersions and their composition (WPI%_R_). In this regard, Figure 6 displays four main regions corresponding to specific morphologies:

- Region I: Particles obtained from dispersions with NPC as the major component, which present a typical wrinkled surface developing along with vacuole formation, and no border detachment, irrespective of c_p_;
- Region II: Particles show a hybrid shape (round shell with dimples) including an internal vacuole and significant delamination. They correspond to suspensions with an intermediate value of WPI%_R_ and low c_p_;
- Region III: Particles present a smooth interface, border detachment, and vacuole formation. They correspond to dispersion with WPI%_R_ ≥ 40% and intermediate/high c_p_;
- Region IV: Particles resulting from dispersions with low c_p_ and WPI as a major component. They show a smooth surface typical of WPI rich samples, similar to samples in Region III, but in contrast, they also exhibit significant delamination and the absence of a vacuole.

## 4. Conclusions

In this work, we aimed at providing an overview of the main shape modifications occurring in WPI/NPC droplets during the evaporation in a pendant configuration. We characterized the droplet diameter reduction (ΔD%) and the final apex height (H_f_/H_max_) as a function of the overall protein concentration (c_p_) in the dispersions and their composition (WPI%_R_). The outcomes highlighted how the different physical phenomena typically affected by WPI%_R_, such as delamination and rigid round shell formation for WPI%_R_ ≥50% and high border adhesivity and skin buckling instability in samples rich in casein micelles, are extremely attenuated or inhibited by the increase of the protein concentration in the initial dispersion. This effect has been interpreted in light of the increase with c_p_ of the NPC volume fraction, in the mixes but also in single WPI suspensions, due to the partial impurity of the commercial powders, which enhance the pinning of the droplet triple line and favor the earlier onset of the sol−gel transition at the air−liquid interface. Such a global overview of the drying process in WPI/NPC mixes allowed for constructing a preliminary phase diagram able to predict the main morphological properties of dried WPI/NPC droplets starting from the initial characteristics of the sample (c_p_, WPI%_R_). This diagram will be much more accurate in the future thanks to ongoing experimental tests and it will also be validated at a larger scale, first by monodisperse drying, and also at the semi-industrial scale. Far from providing an ultimate global picture of the physics of drying in dairy protein mixes, our outcomes represent a preliminary innovative step towards the desired objective of controlling and tuning commercial IMF functional properties using a low-cost, rapid, and simple laboratory scale approach.

## Figures and Tables

**Table 1:** Protein initial volume fraction in WPI/NPC mixes as a function of the protein concentration (c_p_) and sample composition (WPI%_R_).

c_p_ (wt%)	WPI%_R_				
	0	20	50	80	100
6	0.22	0.19	0.14	0.09	0.05
8	0.28	0.24	0.18	0.11	0.06
10	0.33	0.29	0.22	0.14	0.08
12	0.38	0.33	0.26	0.17	0.09
14	0.42	0.37	0.29	0.19	0.11

**Table 2:** Measured values of ΔD% and H_f_/Hmax in drying WPI/NPC droplets with different c_p_ and WPI%_R_. The mean values are presented with their corresponding standard deviations.

c_p_ (wt%)	WPI%_R_									
	0		20		50		80		100	
	∆D%	H/H_0_	∆D%	H/H_0_	∆D%	H/H_0_	∆D%	H/H_0_	∆D%	H/H_0_
6	8.7 ± 1.1	0.40 ± 0.03	8.2 ± 0.6	0.38 ± 0.02	13.2 ± 2.3	0.34 ± 0.01	18.9 ± 2.2	0.28 ± 0.01	21.3 ± 1.8	0.24 ± 0.01
8	6.1 ± 1.6	0.43 ± 0.02	7.8 ± 0.9	0.44 ± 0.02	11.8 ± 1.8	0.41 ± 0.01	18.4 ± 1.1	0.38 ± 0.01	17.5 ± 0.0	0.29 ± 0.01
10	5.8 ± 0.9	0.42 ± 0.02	8.2 ± 1.2	0.41 ± 0.02	9.6 ± 1.0	0.40 ± 0.02	11.0 ± 0.6	0.41 ± 0.01	9.3 ± 0.6	0.39 ± 0.01
12	6.8 ± 1.8	0.52 ± 0.02	5.3 ± 1.3	0.50 ± 0.01	6.2 ± 1.4	0.48 ± 0.02	9.4 ± 1.4	0.45 ± 0.02	9.3 ± 1.5	0.45 ± 0.03
14	4.9 ± 1.7	0.60 ± 0.01	4.6 ± 0.4	0.54 ± 0.01	5.2 ± 0.7	0.53 ± 0.01	7.1 ± 0.7	0.50 ± 0.01	10.8 ± 1.3	0.49 ± 0.02

## Data Availability

Data is contained within the article.
